# Supplementary material for: Unexpected Classes of Aquaporin Channels Detected by Transcriptomic Analysis in Human Brain Are Associated with Both Patient Age and Alzheimer’s Disease Status
Source: Biomedicines. 2023 Mar 3;11(3):770. doi: 10.3390/biomedicines11030770 (PMC10045580; doi:10.3390/biomedicines11030770)
Supplement: Supplementary file 1 [file biomedicines-11-00770-s001.zip › biomedicines-2209202-supplementary.pdf]

Supplementary Tables:

S1. Sub-regions within the HIP, PCx and TCx included in statistical analysis.

HIP Structure Names	PCx Structure Names	TCx Structure Names
CA1 field	Angular Gyrus	Fusiform gyrus
CA2 field	Paracentral lobule (ant.)	Inferior temporal gyrus
CA3 field	Paracentral lobule (post.)	Planum temporale
Dentate Gyrus	Post-central gyrus	Superior temporal gyrus
Subiculum	Precuneus	Temporal pole
	Superior parietal lobule	Transverse gyri
	Supramarginal gyrus	

S2. Gene probe IDs of all human AQP channel genes.

Probe #:	Gene
1054155 and 1054156	*AQP0*
1028728 and 1028729	*AQP1*
1059124 and 1059125	*AQP2*
1059122 and 1059123	*AQP3*
1024273 and 1024274	*AQP4*
1059120 and 1059121	*AQP5*
1059118 and 1059119	*AQP6*
1059116 and 1059117	*AQP7*
1059142 and 1059143	*AQP8*
1059114 and 1059115	*AQP9*
1036971 and 1036972	*AQP10*
1032651 and 1032652	*AQP11*
1031592 and 1031593	*AQP12*

S3. Patient documentation. **a)** Young control group, **b)** Aged control group and **c)** AD group. Males (M), Females (F) and history of traumatic brain injury (TBI).

a)

Donor ID (6)	Age (24-57)	Sex
**H0351.1009**	**57**	**M**
**H0351.1012**	**31**	**M**
**H0351.1015**	**49**	**F**
**H0351.1016**	**55**	**M**
**H0351.2001**	**24**	**M**
**H0351.2002**	**39**	**M**

b)

Donor ID (29)	Age (78-99)	Sex	TBI?	Dementia?
H14.09.078	87	M	N	No Dementia
H14.09.020	95-99	F	N	No Dementia
H14.09.094	78	M	N	No Dementia
H14.09.070	95-99	M	N	No Dementia
H14.09.006	90-94	F	N	No Dementia
H14.09.074	82	F	N	No Dementia
H14.09.072	89	F	N	No Dementia
H14.09.038	78	M	N	No Dementia
H14.09.032	90-94	F	N	No Dementia
H14.09.024	78	M	N	No Dementia
H14.09.060	86	F	N	No Dementia
H14.09.058	88	M	N	No Dementia
H14.09.028	78	M	N	No Dementia
H14.09.062	89	F	N	No Dementia
H14.09.030	90-94	F	N	No Dementia
H15.09.108	90-94	F	N	No Dementia
H14.09.096	84	M	N	No Dementia
H14.09.052	90-94	F	N	No Dementia
H14.09.084	81	M	N	No Dementia
H14.09.102	95-99	F	N	No Dementia
H14.09.014	90-94	M	N	No Dementia
H14.09.050	95-99	M	N	No Dementia
H14.09.090	89	F	N	No Dementia
H14.09.046	95-99	M	N	No Dementia
H15.09.104	90-94	F	N	No Dementia
H14.09.080	87	M	N	No Dementia
H15.09.106	86	M	N	No Dementia
H14.09.076	78	F	N	No Dementia
H14.09.016	81	M	N	No Dementia

c)

Donors (11)	Age (79-100+)	Sex	TBI?	Dementia?	Diagnosis
			**BRAAK**		
			**STAGE 1**		
H14.09.002	90-94	M	N	Dementia	Probable AD

H14.09.022	88	M	N	Dementia	Probable AD
			**STAGE 2**		
H15.09.110	82	F	N	Dementia	Probable AD
H14.09.018	79	M	N	Dementia	Probable AD
			**STAGE 3**		
H14.09.054	89	M	N	Dementia	Probable AD
			**STAGE 5**		
H14.09.034	88	M	N	Dementia	Probable AD
H14.09.068	85	M	N	Dementia	Probable AD
			**STAGE 6**		
H14.09.098	86	M	N	Dementia	Probable AD
14.09.044	87	F	N	Dementia	Probable AD
H14.09.086	95-99	F	N	Dementia	Probable AD
H14.09.040	85	M	N	Dementia	Probable AD

S4. Supervised cluster analysis comparing *AQP* gene expression profiles between HIP, PCx and TCx. AQP probes defining component 1 and component 2.

Probe	Gene	Loading
**Component 1 defining probes**		
1059114	*AQP9*	0.99
1032651	*AQP11*	0.16
**Component 2 defining probes**		
1059119	*AQP6*	0.37
1059122	*AQP3*	-0.35
1059121	*AQP5*	-0.33
1031593	*AQP12*	0.32
1059117	*AQP7*	0.27
1059123	*AQP3*	-0.26
1059115	*AQP9*	0.22
1059143	*AQP8*	0.21
1059125	*AQP2*	0.20
1032652	*AQP12*	0.20
1024273	*AQP4*	-0.19
1031592	*AQP12*	0.17
1059124	*AQP2*	0.17
1024274	*AQP4*	-0.15
1032651	*AQP11*	0.15
1054156	*AQP0*	0.14
1059120	*AQP5*	0.14
1054155	*AQP0*	0.12
1028728	*AQP1*	0.08
1028729	*AQP1*	0.06
1059116	*AQP7*	0.01
1059114	*AQP9*	-0.002
